# Correlation Between Myopia Degree and Postoperative Dry Eye Disease in Keratorefractive Lenticule Extraction Surgery

**DOI:** 10.7759/cureus.100163

**Published:** 2025-12-26

**Authors:** Chia-Yi Lee, Shun-Fa Yang, Yu-Ling Chang, Jing-Yang Huang, Feng-Ming Yeh, Chao-Kai Chang

**Affiliations:** 1 Institute of Medicine, Chung Shan Medical University, Taichung, TWN; 2 Department of Pediatric Medicine, Cathay General Hospital, Taipei, TWN; 3 Department of Medical Research, Chung Shan Medical University Hospital, Taichung, TWN; 4 Department of Optometry, Yuanpei University of Medical Technology, Hsinchu, TWN; 5 Department of Ophthalmology, Nobel Eye Institute, Taipei, TWN

**Keywords:** dry eye disease, keratorefractive lenticule extraction, myopia, superficial keratitis, visumax 800

## Abstract

Purpose: This study aimed to investigate the relationship between myopia degree and the rate of postoperative dry eye disease (DED) in participants who received keratorefractive lenticule extraction (KLEx) surgery.

Methods: A retrospective cohort study was conducted, and participants who received KLEx surgery were included. After selection, a total of 95 and 76 eyes were classified into the low myopia (LM) and high myopia (HM) groups, respectively. The main outcomes are the postoperative tear break-up time (TBUT), Schirmer test, ocular surface staining, and DED-related symptoms. The independent t-test and generalized linear model were used for the statistical analysis of the present study.

Results: The postoperative uncorrected distance visual acuity (UDVA) and spherical equivalent (SE) were significantly better in the LM group (all p<0.001). Regarding the postoperative DED parameters, the TBUT and Schirmer test values were significantly higher in the LM group compared to those in the HM group during the whole follow-up period (all p<0.05). The rate of superficial keratitis and multiple DED symptom were significantly higher in the HM group than the LM group one month postoperatively (both p<0.05). Advanced age correlated with poor DED sign, and female sex correlated with poor DED symptom in the LM group (both p<0.05). On the other hand, both advanced age and female sex were associated with poor DED signs and symptoms in the HM group (all p<0.05).

Conclusion: HM may have a certain correlation with higher DED incidence and severity for participants who received KLEx surgery.

## Introduction

Keratorefractive surgeries were introduced for the correction of several refractive errors involving myopia, presbyopia, astigmatism, and hyperopia in the early 1990s [1,2]. In the recent 10 years, the keratorefractive lenticule extraction (KLEx) surgery has gained recognition in comparison with other keratorefractive surgeries because of the small corneal wound and the small percentage of postoperative dry eye disease (DED) [3,4]. The second-generation KLEx surgery, an innovation of the first-generation KLEx surgery, was administered for over two years, and the postoperative uncorrected distance visual acuity (UDVA) can attain 20/20 in most individuals [5]. Besides, the postoperative residual refractive error of second-generation KLEx surgery was below 0.50 diopter (D) in most individuals according to the earlier study [6].

Although the efficiency and predictability of the first- and second-generation KLEx surgeries are acceptable [7], severe postoperative complications could still present [8]. In the previous publication, the corrected distance visual acuity (CDVA) loss may present after the KLEx surgeries [8,9]. In addition to CDVA loss, the prominent residual refractive error, principally myopia and astigmatism, may reduce the postoperative UDVA of the KLEx surgery, in which an enhancement approach may be advocated to rescue the postoperative UDVA [3,10]. In addition to the above complications, superficial keratitis is a postoperative complication of KLEx surgeries, although the probability is low [11]. Furthermore, postoperative DED can influence the outcome of keratorefractive surgery like laser in situ keratomileusis [12].

Some factors could influence the chance of postoperative complications of keratorefractive surgeries in previous research [13]. Old age is a predisposing factor for postoperative DED in patients who received laser in situ keratomileusis [14]. In addition, individuals with high myopia would contribute to unstable postoperative visual recovery after the KLEx surgery [10]. Nevertheless, there was little research to evaluate the influence of myopia degree on postoperative DED of KLEx surgery. Because myopia correlates with inflammation status, which is a crucial factor for DED development [15,16], such a possibility may exist which need survey. On the other hand, the pre-existing risk factor of DED (i.e., old age) may contribute to the unstable postoperative condition of KLEx surgery in clinical practice; thus, this issue may also be investigated.

As a consequence, the purpose of the present study is to investigate the potential association between myopia degree and the incidence of postoperative DED of KLEx surgery. The primary outcomes compared in the present study are the DED signs and symptoms, which included the tear break-up time (TBUT), Schirmer test, ocular surface staining, and DED symptoms; the changes of these DED parameters after KLEx surgery between the different groups would be analyzed separately. The secondary objectives, which mean the risk factors for poor DED outcomes, were also evaluated in the present study.

## Materials and methods

Ethics declaration

The present study adhered to the principles of the 1964 Declaration of Helsinki and its subsequent amendments. Ethical approval was obtained from the Institutional Review Board of National Changhua University of Education (approval number: NCUEREC-113-063). Owing to the retrospective nature of the present study, the requirement for written informed consent was waived. 

Subject selection

A retrospective cohort study was administered in the Nobel Eye Institute, Taipei, Taiwan, a clinic group that principally operates keratorefractive and cataract surgeries. Participants with the following criteria were chosen into our study population: (1) myopia status with sphere power ranging from -1.00D to -9.00D, (2) astigmatism less than -5.00D cylinder power, (3) received second-generation KLEx surgery in any department of the Nobel Eye Institute, and (4) received post-operation follow-up for at least three months in any department of the Nobel Eye Institute. On the contrary, the following exclusion criteria was administered to standardize the general features of our study population: (1) age lower than 20 years, (2) age older than 55 years, (3) the presence of previous uveitis, glaucoma, retinal diseases except retinal break, ptosis, keratoconus, subclinical keratoconus status, corneal opacity, complicated cataract, and optic neuropathy, and (4) the receipt of monovision (planned residual myopia) intervention. Subsequently, the study population was classified into the low myopia (LM) group and the high myopia (HM) group by the spherical equivalent (SE) refractive error of -6.00D. The threshold of LM and HM is according to the International Myopia Institute standard [17]. Importantly, only the right eye of each participant was enrolled in the present study. After the whole selection procedure, there were 95 and 79 eyes that received second-generation KLEx surgery in the LM group and the HM group, respectively.

Surgery technique

All second-generation KLEx surgeries were accomplished by two experienced refractive surgeons (C.-K.C. and C.-Y.L.) using a femtosecond laser platform (VisuMax 800, Carl Zeiss, Jena, Germany). About surgical indexes, the optic zone ranged from 5.5 to 6.9 mm with a 3-mm incision positioned at 105°, and the angle kappa was calculated by the advanced optical biometry (IOLMaster 700, Carl Zeiss, Jena, Germany). Besides, centration was refined using the coaxial corneal light-reflex method and corneal topography. After centration, the corneal surface was fixated with a suction ring, and the laser was activated upon the surgeon's confirmation. Following lenticule creation via laser, the upper and lower interfaces were dissected via dissector, and the lenticule was removed with specialized forceps. Postoperatively, levofloxacin and prednisolone eye drops were prescribed for one week, followed by sulfamethoxazole and fluorometholone for three weeks. Preservative-free artificial tears were used for two weeks, and regular artificial tears were used for an additional two months.

Ophthalmic data

All participants undergoing second-generation KLEx surgery completed the same standardized examinations at every branch of the Nobel Eye Institute. The demographic data and DED-related symptoms (e.g., dryness, foreign body sensation, grittiness, burning) were recorded in the first consultation. Preoperative assessments included CDVA, cycloplegic refraction (i.e., cycloplegic sphere and cylinder powers) measured with an autorefractor (KR-8900, Topcon, Tokyo, Japan), as well as central corneal thickness, angle kappa, topographic cylinder power, and pupil size obtained from a corneal topographer (TMS-5, Tomey Corporation, Nagoya, Japan). Angle kappa was additionally verified using optical biometry (IOLMaster 700). The slit-lamp biomicroscope was applied to examine the external eye, and superficial keratitis would be observed and recorded if it existed. About the DED-related exams, the TBUT and Schirmer test without topical anesthesia were performed before KLEx surgery. For the TBUT, the fluorescein strip was put onto the conjunctival surface, and then the participant was asked to close their eye for five seconds. After that, the participant was asked to open their eye and hold it, and the time from opening their eye to the emergence of the black spot on the corneal surface was recorded by the physician. For the Schirmer test, a Schirmer strip was placed in the lateral third of the palpebral conjunctiva of the lower eyelid. Then the participant was asked to close their eye and rest for five minutes, the Schirmer strip was removed, and the length of the tear-induced wetting zone was recorded. In addition to all the above data, surgical parameters, including optic zone (OZ) diameter and residual stromal thickness (RST), were obtained from the surgical record. Postoperative evaluations consisted of UDVA, refractive measurements, slit-lamp examination, TBUT, and Schirmer test using the same instruments, and CDVA was documented three months after the KLEx procedure. The presence of DED-related symptoms was also recorded after the KLEx surgery if it existed. In the present study, SE was defined as the spherical value plus half of the cylindrical correction, and the reported angle kappa value represents the average derived from both topographic and biometric measurements. Clinical data were collected at the preoperative visit and at one day, one week, one month, and three months following KLEx surgery.

Statistical analysis

The statistical analyses in the present study were performed using IBM SPSS Statistics for Windows, Version 20.0 (IBM Corp., Armonk, New York, United States). The distribution of all variables was assessed with the Shapiro-Wilk test, which confirmed the normality for every parameter (all p>0.05). Moreover, statistical power was calculated with G*Power 3.1.9.2 (Heinrich-Heine-Universität Düsseldorf, Düsseldorf, Germany), yielding a power of 0.97 with an alpha level of 0.05 and a medium effect size. Because all cases were followed by one experienced physician (C.-Y.L.) after the KLEx surgery, the inter-observer variability may be small in the present study. Descriptive statistics were used to summarize the demographic characteristics, CDVA, cycloplegic refraction, topographic measurements, DED-related parameters, and surgical parameters across the two groups. Depending on the variable characteristic, the chi-squared test or independent t-test was applied to compare the preoperative characteristics between the LM and HM groups. The independent t-test was also used to evaluate postoperative UDVA, SE, CDVA, TBUT, and Schirmer test results at each follow-up point between the LM and HM groups. On the other hand, the chi-squared test was applied to evaluate the number of postoperative superficial keratitis and DED-related symptoms between the LM and HM groups. Multiple DED symptoms referred to the presence of more than three DED-related symptoms. In the next step, we separated the participants into those with poor DED sign condition (TBUT lower than 10 seconds, Schirmer test lower than 10 mm, or the presence of superficial keratitis three months after the KLEx surgery) and poor DED symptom condition (suffered from more than three DED-related symptoms three months after the KLEx surgery). Then the generalized linear model was administered to evaluate the correlation between advanced age (older than 45 years old), female sex, and large OZ (larger than 6.8 mm) with the poor DED sign condition as well as the poor DED symptom condition in both groups. The odds ratio (OR) and corresponding 95% confidence interval (CI) were obtained via the generalized linear model. A p-value of <0.05 was regarded as statistically significant, a p-value exceeding 0.999 was reported as p>0.999, and those p-values below 0.001 were reported as p<0.001 in the present study.

## Results

The baseline characteristics between the two groups are shown in Table 1. The mean age was 36.21±10.33 years old and 35.86±10.47 years old in the LM and HM groups, respectively, and the age difference did not achieve a significant difference between the two groups (p=0.825). The sex distribution also represented an insignificant difference between the LM and HM groups (p=0.877). About the ophthalmic parameters, the sphere power, cylinder power, SE, and topographic cylinder all demonstrated significantly higher values in the HM group compared to the LM group (all p<0.001), while all the DED parameters illustrated no significant difference between the two groups (all p>0.05). Besides, the RST was significantly lower in the HM group than the LM group (p=0.002).

**Table 1:** Baseline characteristics between the two groups The chi-squared test and independent t-test were used for the statistical analysis. * denotes a significant difference between the two groups. CDVA: corrected distance visual acuity; CCT: central corneal thickness; D: diopter; DED: dry eye disease; HM: high myopia; LM: low myopia; N: number; OZ: optic zone; RST: residual stromal thickness; SE: spherical equivalent; TBUT: tear break-up time

Features	LM group (N=95)	HM group (N=79)	Test statistic	P-value
Age (year)	36.21±10.33	35.86±10.47	0.221	0.825
Sex				
Male	39	31	0.059	0.877
Female	56	48		
CDVA (LogMAR)	0.00±0.02	0.00±0.03	0.000	0.999
Cycloplegic refraction (D)				
Sphere	-3.62±1.19	-7.75±1.67	18.432	<0.001*
Cylinder	-1.08±0.36	-1.42±0.69	3.955	<0.001*
SE	-4.16±0.73	-8.46±1.18	28.210	<0.001*
Topographic cylinder (D)	-1.35±0.52	-1.80±0.83	4.184	<0.001*
CCT (μm)	542.72±26.87	543.17±30.22	-0.104	0.917
Angle kappa	0.15±0.10	0.12±0.11	1.883	0.061
Pupil diameter (mm)	4.32±1.01	4.28±0.90	0.273	0.785
TBUT (second)	11.26±1.88	10.72±1.76	1.942	0.054
Schirmer test (mm)	14.68±2.36	14.06±2.29	1.749	0.082
Superficial keratitis (N)	0	0	0.000	0.999
>3 DED-related symptoms (N)	2	4	1.134	0.413
OZ (mm)	6.38±0.37	6.26±0.53	1.698	0.092
RST (μm)	294.96±10.87	288.72±10.66	3.803	0.002*

The UDVA one day after KLEx surgery was 0.17±0.07 and 0.27±0.13 in the LM and HM groups, respectively, and the LM group had a significantly better UDVA (p<0.001). At the final visit (i.e., three months postoperatively), the LM group still showed a significantly better UDVA than the HM group (0.03±0.02 versus 0.06±0.04; p<0.001). On the other hand, the LM group presented a lower SE than the HM group one day postoperatively (-0.16±0.20D versus -0.38±0.43D; p<0.001). The SE three months postoperatively was -0.09±0.06D and -0.33±0.17D in the LM and HM groups, respectively, and the SE was still lower in the LM group than the HM group (p<0.001). For the CDVA, the CDVA three months postoperatively was significantly better in the LM group (0.00±0.05) compared to the HM group (0.02±0.06) (p=0.018) (Table 2).

**Table 2:** Postoperative uncorrected distance visual acuity and spherical equivalent between the two groups The independent t-test was used for the statistical analysis. * denotes a significant difference between the two groups. CDVA: corrected distance visual acuity; D: diopter; HM: high myopia; LM: low myopia; SE: spherical equivalent; UDVA: uncorrected distance visual acuity

Outcome	LM group	HM group	Test statistic	P-value
UDVA (LogMAR)				
1 day	0.17±0.07	0.27±0.13	-6.137	<0.001*
1 week	0.08±0.05	0.18±0.10	-8.087	<0.001*
1 month	0.03±0.03	0.12±0.06	-12.131	<0.001*
3 months	0.03±0.02	0.06±0.04	-6.065	<0.001*
SE (D)				
1 day	-0.16±0.20	-0.38±0.43	4.186	<0.001*
1 week	-0.10±0.07	-0.30±0.22	7.760	<0.001*
1 month	-0.11±0.06	-0.26±0.15	8.350	<0.001*
3 months	-0.09±0.06	-0.33±0.17	11.945	<0.001*
CDVA				
3 months	0.00±0.05	0.02±0.06	-2.399	0.018*

Regarding the postoperative DED parameters, the TBUT and Schirmer test values were significantly higher in the LM group compared to those in the HM group during the whole follow-up period (all p<0.05). The rate of superficial keratitis and multiple DED symptom were significantly higher in the HM group than the LM group one month postoperatively (both p<0.05), and the ratio became similar between the two groups after three months (both p>0.05) (Table 3).

**Table 3:** Postoperative dry eye parameters between the two groups The chi-squared test and independent t-test were used for the statistical analysis. * denotes a significant difference between the two groups. DED: dry eye disease; HM: high myopia; LM: low myopia; TBUT: tear break-up time

Parameter	LM group	HM group	Test statistic	P-value
TBUT (second)				
1 month	9.93±1.64	7.23±1.59	10.963	<0.001*
3 months	10.89±1.71	8.08±1.66	10.936	<0.001*
Schirmer test (mm)				
1 month	13.97±2.31	13.21±2.25	2.186	0.030*
3 months	14.52±2.44	13.68±2.35	2.299	0.023*
Superficial keratitis				
1 month	2	10	7.481	0.013*
3 months	0	3	3.671	0.055
>3 DED-related symptoms				
1 month	6	13	4.559	0.029*
3 months	4	9	3.218	0.066

Advanced age correlated with poor DED sign condition (p=0.008), and the female sex correlated with poor DED symptom condition (p=0.029) in the LM group (Table 4).

**Table 4:** Factors for poor dry eye condition in the low myopia group The generalized linear model was used for the statistical analysis. * denotes a significant correlation between the parameter and DED condition. CI: confidence interval; DED: dry eye disease; OR: odds ratio; OZ: optic zone

Parameter	OR	95% CI	P-value
Sign			
Advanced age	1.07	1.02-1.12	0.008*
Female sex	1.04	0.97-1.13	0.194
Large OZ	1.02	0.95-1.09	0.776
Symptom			
Advanced age	1.04	0.98-1.10	0.137
Female sex	1.05	1.01-1.09	0.029*
Large OZ	1.02	0.93-1.07	0.643

On the other hand, advanced age, female sex, and large OZ were associated with poor DED sign condition (all p<0.05), and advanced age and female sex were associated with poor DED symptom condition (both p<0.05) in the HM group (Table 5).

**Table 5:** Factor for poor dry eye condition in the high myopia group The generalized linear model was used for the statistical analysis. * denotes a significant correlation between the parameter and DED condition. CI: confidence interval; DED: dry eye disease; OR: odds ratio; OZ: optic zone

Parameter	OR	95% CI	P-value
Sign			
Advanced age	1.15	1.06-1.25	<0.001*
Female sex	1.10	1.04-1.16	0.001*
Large OZ	1.06	1.01-1.11	0.016*
Symptom			
Advanced age	1.16	1.08-1.24	<0.001*
Female sex	1.13	1.06-1.20	<0.001*
Large OZ	1.03	0.98-1.08	0.258

## Discussion

In the present study, the participants with HM represented a poorer postoperative TBUT, Schirmer test result, ocular surface condition, and DED-related symptoms than the participants with LM after KLEx surgery. Moreover, more factors are correlated with poor postoperative DED condition in the HM group than in the LM group. Besides, the participants with HM demonstrated a worse postoperative UDVA and SE than the participants with LM after the KLEx surgery.

HM has become a major concern in ophthalmic practice and has been connected to numerous ocular complications in prior research [18]. Individuals with prominent myopia exhibit an increased likelihood of developing open-angle glaucoma, and these two conditions share overlapping pathogenic mechanisms [19]. Furthermore, retinal detachment occurs more frequently in the high-myopia population [20]. Previous literature have also indicated that HM contributes to an elevated risk of cataract formation [21] and affected patients commonly undergo cataract surgery at a comparatively young age, often around their 40s [22]. Among individuals with extremely HM, defined as a spherical equivalent greater than -10.00D, the incidence of pediatric retinal detachment is markedly increased, and surgical outcomes tend to be less favorable compared with those with moderate HM [23]. At the molecular level, myopia is an inflammatory disease in which the HM is featured with a high baseline inflammatory status, such as higher neutrophil-to-lymphocyte ratios, than normal individuals [15]. In addition, inflammatory cytokines like the matrix metalloproteinases and interleukins showed significantly higher concentrations in individuals with HM [24]. DED is an inflammatory ocular disease that presents with unstable tear film and loss of homeostasis and can be triggered by refractive surgery [25]. In the previous study, the concentration of interleukin and tumor necrosis factor would elevate in individuals with DED [16]. Besides, anti-inflammatory agents such as steroids and immunosuppressants can be administered for the management of advanced DED [26]. Because of the high baseline inflammatory status in HM individuals, we propose that the presence of HM may aggravate the post-KLEx surgery DED. This concept was supported by the findings of the present study at least partially.

In the present study, the HM population demonstrated a significantly lower TBUT, Schirmer test results, ocular surface staining, and DED-related symptoms compared to the LM population after the arrangement of KLEx surgery. In the previous study, the HM status was correlated with the relatively unstable postoperative UDVA and myopic regression [10]. According to another study, the chance of under-correction after KLEx surgery was significantly more frequent in the HM population compared to their LM counterpart [27]. Nevertheless, there was a rare study investigating the correlation of myopia degree and the incidence and severity of post-KLEx surgery DED. To our knowledge, the findings of the present study may be a preliminary experience that represents the positive correlation between the preoperative myopia degree and the postoperative DED development and severity. In addition, the baseline data, including the DED parameters, between the LM and HM groups in the present study were similar. Thus, the influence of baseline factors may be little. Furthermore, the postoperative care protocols of KLEx surgery among all the Nobel Eye Institute clinics are identical, so the impact of different postoperative medications on our study population may be minimal. As a consequence, the higher degree of myopia might be an independent risk factor for the postoperative DED of KLEx surgery. The sign of DED, including the TBUT and Schirmer test result, demonstrated significantly lower values in the HM group than the LM group throughout the postoperative period, which indicates the general effect of HM status on the ocular surface. On the other hand, the superficial keratitis incidence and multiple DED-related symptom numbers were significantly higher in the HM group than in the LM group one month postoperatively. This phenomenon may indicate that the influence of HM on post-KLEx surgery DED is not severe enough to serve as a relative contraindication. In a clinical situation, the moderate difference of TBUT and Schirmer test result (10-20% difference) and the absolute value of these two DED parameters (more than 5) between groups may not contribute to a significant impact of postoperative care; thus, the DED condition in the HM group may be acceptable compared to the LM group.

In the sensitive analysis, advanced age and female sex are partially correlated with poor DED sign and symptom conditions in those with LM status, while advanced age, female sex, and large OZ are associated with poor DED sign and symptom conditions in those with HM status. Concerning the known risk factor for DED development, advanced age is an established risk factor for DED development, in which the incidence of DED was significantly higher in the old population than the young population in the previous literature [25]. Female sex is another known risk factor for the development of DED, and the previous study demonstrated a significantly more severe DED symptom in the female population compared to their male counterpart [28]. As a consequence, it is reasonable that advanced age and female sex contribute to the poor DED sign and symptom condition after KLEx surgery in participants with HM. On the other hand, advanced age and female sex only partially relate to the poor DED sign and symptom condition after KLEx surgery in participants with LM. There was no research to report this phenomenon. The discordance effect of the above factors on postoperative DED conditions between the LM and HM groups further states that HM is a condition vulnerable to DED damage, especially for those receiving refractive surgery, and the high baseline inflammation may be the main etiology for this circumstance. Large OZ is not a general risk factor of DED, but it is significantly correlated with poor DED sign condition in the HM group. We think the possible mechanism is that the additional laser energy for the creation of large OZ during KLEx surgery could contribute to corneal edema and inflammatory status; thus, DED sign under such conditions may increase in the vulnerable population like HM individuals. 

Concerning the postoperative outcomes of KLEx surgery between the LM and HM groups of the present study, the postoperative UDVA was better in the LM group than the HM group throughout the study period, and the visual recovery was slower in the HM group than the LM group, in which the UDVA became stable in the LM group one month after the KLEx surgery. These results may correspond to the previous experience where the HM population had a worse postoperative visual acuity after keratorefractive surgery [27]. Also, the presence of DED may affect visual quality; thus, the postoperative UDVA of the HM group may also be reduced by DED presence. On the other hand, a higher amount of SE was found in participants with HM compared to the participants with LM after the KLEx surgery, and the fluctuation of SE was more prominent in the HM group compared to the LM group during the postoperative interval. HM also represented a higher amount of residual refractive error in the previous publication [10], and it is reasonable that the HM group in the present study demonstrated more SE after KLEx surgery than the LM group. The CDVA value, which indicates the safety of the keratorefractive surgery, was significantly worse in the HM group than in the LM group three months after the KLEx surgery. Considering all the above results, the participants with HM may have a worse postoperative outcome than the participants with LM. Nevertheless, both the UDVA and CDVA values of the HM group were also near 20/20 three months postoperatively, and the visual outcomes of the HM group were also comparable to the postoperative UDVA of the general population in the previous study [6]. Consequently, the HM status may cause unstable postoperative visual acuity and high DED incidence after KLEx surgery, but the general outcomes are acceptable.

There are some limitations in the present study. Firstly, the retrospective nature of the present study would reduce the homogeneity of the study population, although the baseline characteristics were all similar between the two groups, and the postoperative topography was also absent due to the same reason. Secondly, the participant numbers were relatively insufficient, with only 174 eyes included, despite the fact that the statistical power reached a fair amount. In addition, we did not administer the standard DED symptom evaluation tool, like the Ocular Surface Disease Index chart, to evaluate our participants, which may cause the inaccuracy of result production. Besides, measurement bias (e.g., lack of masking), the limited control of confounding variables, selection bias (only right eye inclusion, monovision exclusion), the absence of pre-registration, the mask effect of postoperative artificial tear supplement on DED signs and symptoms, sample size discrepancy, and the potential effect of RST difference could also influence the integrity of our analyses and results. Finally, all the participants in the present study were Han Taiwanese; thus, the external validity of the present study may be insufficient.

## Conclusions

The presence of HM may have a certain correlation with the higher incidence of postoperative DED development after KLEx surgery than the LM population. Furthermore, the number of predisposing factors for poor postoperative DED status was higher in the HM participants compared to their LM counterparts. Consequently, preoperative DED management may be recommended for HM patients scheduled for KLEx surgery and with risk factors for postoperative DED.* *Further large-scale prospective studies to investigate whether the high postoperative DED rate and severity in the HM population can be reversed by the preoperative DED management are mandatory.

## References

[1] Ang M, Gatinel D, Reinstein DZ, Mertens E, Alió Del Barrio JL, Alió JL (2021). Refractive surgery beyond 2020. Eye (Lond).

[2] Zou H, Wei X, Li L (2024). Comparison of objective visual quality between SMILE and FS-LASIK in moderate-to-high myopia. Front Med (Lausanne).

[3] Alió Del Barrio JL, Vargas V, Al-Shymali O, Alió JL (2017). Small incision lenticule extraction (SMILE) in the correction of myopic astigmatism: outcomes and limitations - an update. Eye Vis (Lond).

[4] Lee JK, Chuck RS, Park CY (2015). Femtosecond laser refractive surgery: small-incision lenticule extraction vs. femtosecond laser-assisted LASIK. Curr Opin Ophthalmol.

[5] Lee CY, Lian LB, Chen HC, Huang CT, Huang JY, Yang SF, Chang CK (2024). The outcomes of first-generation (Visumax 500) and second-generation (Visumax 800) keratorefractive lenticule extraction surgeries for astigmatism. Sci Rep.

[6] Saad A, Klabe K, Kirca M, Kretz FA, Auffarth G, Breyer DR (2024). Refractive outcomes of small lenticule extraction (SMILE) Pro® with a 2 MHz femtosecond laser. Int Ophthalmol.

[7] Lee CY, Lian IB, Chen HC, Huang CT, Huang JY, Yang SF, Chang CK (2024). The efficiency, predictability, and safety of first-generation (Visumax 500) and second-generation (Visumax 800) keratorefractive lenticule extraction surgeries: real-world experiences. Life (Basel).

[8] Zhong Y, Li M, Han T, Fu D, Zhou X (2021). Four-year outcomes of small incision lenticule extraction (SMILE) to correct high myopic astigmatism. Br J Ophthalmol.

[9] Reinstein DZ, Archer TJ, Potter JG, Gupta R, Wiltfang R (2023). Refractive and visual outcomes of SMILE for compound myopic astigmatism with the VISUMAX 800. J Refract Surg.

[10] Lee CY, Jeng YT, Chao CC, Lian IB, Huang JY, Yang SF, Chang CK (2024). Refraction and topographic risk factors for early myopic regression after small-incision lenticule extraction surgery. Sci Rep.

[11] Lee CY, Chen HC, Yang SF, Hsueh YJ, Huang JY, Chang CK (2025). The effect of preoperative total antioxidant capacity on short-term outcomes after kerato-lenticule extraction surgery. Sci Rep.

[12] Tamimi A, Sheikhzadeh F, Ezabadi SG (2023). Post-LASIK dry eye disease: a comprehensive review of management and current treatment options. Front Med (Lausanne).

[13] Nair S, Kaur M, Sharma N, Titiyal JS (2023). Refractive surgery and dry eye - an update. Indian J Ophthalmol.

[14] Shoja MR, Besharati MR (2007). Dry eye after LASIK for myopia: incidence and risk factors. Eur J Ophthalmol.

[15] Xu R, Zheng J, Liu L, Zhang W (2023). Effects of inflammation on myopia: evidence and potential mechanisms. Front Immunol.

[16] Kumar NR, Praveen M, Narasimhan R (2023). Tear biomarkers in dry eye disease: progress in the last decade. Indian J Ophthalmol.

[17] Flitcroft DI, He M, Jonas JB (2019). IMI - defining and classifying myopia: a proposed set of standards for clinical and epidemiologic studies. Invest Ophthalmol Vis Sci.

[18] Morgan IG, Ohno-Matsui K, Saw SM (2012). Myopia. Lancet.

[19] Ma F, Dai J, Sun X (2014). Progress in understanding the association between high myopia and primary open-angle glaucoma. Clin Exp Ophthalmol.

[20] Haarman AE, Enthoven CA, Tideman JW, Tedja MS, Verhoeven VJ, Klaver CC (2020). The complications of myopia: a review and meta-analysis. Invest Ophthalmol Vis Sci.

[21] Shah R, Vlasak N, Evans BJ (2024). High myopia: reviews of myopia control strategies and myopia complications. Ophthalmic Physiol Opt.

[22] Du Y, Meng J, He W, Lu Y, Zhu X (2023). Challenges of refractive cataract surgery in the era of myopia epidemic: a mini-review. Front Med (Lausanne).

[23] Wang NK, Chen YP, Lai CC (2009). Paediatric retinal detachment: comparison of high myopia and extreme myopia. Br J Ophthalmol.

[24] Zhang J, Kamoi K, Zong Y, Yang M, Zou Y, Ohno-Matsui K (2025). Inflammation and immune pathways in myopia: an overview on pathomechanisms and treatment prospects. Clin Rev Allergy Immunol.

[25] Clayton JA (2018). Dry eye. N Engl J Med.

[26] Mohamed HB, Abd El-Hamid BN, Fathalla D, Fouad EA (2022). Current trends in pharmaceutical treatment of dry eye disease: a review. Eur J Pharm Sci.

[27] Lee CY, Yang SF, Chen HC, Lian IB, Huang CT, Huang JY, Chang CK (2024). The risk factors for myopia undercorrection in second-generation (Visumax 800) keratorefractive lenticule extraction surgery: a retrospective case-control study. Diagnostics (Basel).

[28] Rouen PA, White ML (2018). Dry eye disease: prevalence, assessment, and management. Home Healthc Now.

